# Effect of immunization against ox-LDL with two different antigens on formation and development of atherosclerosis

**DOI:** 10.1186/1476-511X-6-32

**Published:** 2007-11-24

**Authors:** Sedigheh Asgary, Salb-Ali Saberi, Shirin Azampanah

**Affiliations:** 1 Basic sciences department, Isfahan Cardiovascular Research Center, Isfahan University of Medical Sciences, Isfahan, Iran; 2 Isfahan University of Medical Sciences. Isfahan Cardiovascular Research Center a WHO Collaborating Center for Research and Training in Cardiovascular Diseases Control, Prevention for Cardiac Patients in EMRO

## Abstract

**Background:**

Several studies were pointed to oxidized LDL (ox-LDL) as one of the main immunogenes which have important roles in primary lesions of atherosclerosis. In this study, by immunization against ox-LDL with two different antigens in an animal model (rabbit) and consideration of its effect on two different dietary regimens; we tried to clear relation between immune system and atherosclerosis.

**Methods:**

LDL was isolated from hypercholesterolemic rabbits plasma and oxidized with MDA or Cu^++^. Rabbits were divided to three groups and immunized with MDA-LDL or Cu-LDL or phosphate-buffer (PBS) as a control group. Immunization was repeated after 2, 4, 6, and 8 weeks and concentration of antibodies against ox-LDL was measured in each stage. After immunization, rabbits in each group were divided to two subgroups based on the dietary regimen (fed normal or high cholesterol diet). At the beginning and the end of the study, biochemical factors were measured. Also, fatty streaks in aorta and left and right coronary arteries evaluated.

**Results:**

Immunization with Cu^2+^-LDL and MDA-LDL induced statistically significant antibodies against ox-LDL. In hypercholesterolemic rabbits immunized with MDA-LDL the level of cholesterol, LDL-cholesterol, triglyceride, fasting blood sugar and fatty streak lesions in aorta and right coronary arteries were significantly decreased as compared with non-immunized high-cholesterol group. Immunization with Cu^2+^-LDL in hypercholesterolemic rabbits significantly decreased triglyceride, fasting blood sugar, cholesterol and CRP. No significant differences were detected in the fatty streak lesions in this group as compared with non-immunized high-cholesterol diet. In groups under normal diet immunized with MDA-LDL or Cu^2+^-LDL no significant effect on biochemical factors and atherosclerotic lesions were observed.

**Conclusion:**

This study indicates that although the effect of produced antibodies in several methods and different dietary regimens is different, immunization against ox-LDL is antiatherogenic.

## Background

In the recent studies, relation between immune system and atherosclerosis has been investigated [1-3]. It is clear that this system can be effective on severity of atherosclerosis through different immunogenic mechanisms [4-6]. Several studies were pointed to oxidized LDL (ox-LDL) as one of the main immunogenes which have important roles in primary lesions of atherosclerosis [5,6]. Little changes in LDL strongly converted it to immunogenic particle. ox-LDL is a toxic particle that stimulates several cellular and humeral responses [7]. The existence of activated macrophages against ox-LDL in atherosclerotic lesions is a marker for primary cellular responses [8]. Different types of antibodies against ox-LDL were recognized in blood stream. These antibodies are measured in both human and animal models [9-12] bind to ox-LDL epitopes and cause atherosclerosis. As, hypercholesterolemic rabbits immunized with homologous, ox-LDL have a markedly reduced neointimal area after balloon injury despite sever hypercholesterolemia, then immunization against atherosclerosis also is a target to prevent restenosis and further investigation in this field is warranted [13].

However, the atherogenic or athero-protective role of immune system in atherosclerosis is not clearly demonstrated. In this study, by immunization against ox-LDL with two different antigens in an animal model (rabbit) and consideration of its effect on two different dietary regimens (fed normal or high cholesterol diet); we tried to clear relation between immune system and atherosclerosis.

## Methods

### Isolation and oxidation of LDL

LDL was isolated by ultracentrifuge from healthy New Zealand white rabbits with diet-induced hypercholesterolemia and modified with MDA and Cu^2+^. LDL (1.019 < d < 1.063 g/mL) was isolated by sequential ultracentrifugation in the presence of antioxidants and antiproteolytic agents. The density was adjusted to 1.022 g/mL with KBr, then plasma was centrifuged at 65000 rpm for 4 hours at 10°C using a Beckman L7-65 ultracentrifuge with a VTi 65.2 rotor. The supernatant was pipetted off, the density of the infranatant was adjusted to 1.063 g/mL with KBr, and the LDL fraction was isolated by centrifugation at 44000 rpm for 22 hours using a 50.3 rotor. LDL was then extensively dialyzed against EDTA-free phosphate buffer (PBS) and sterile filtered [14].

Cu-LDL was generated by incubating 160 μg LDL per mL PBS, pH 7.35, with 5 μmol/L CuSO4 at 37°C [14,15]. Oxidation of LDL by MDA is carried out according to Foglemen procedure [16].

### Immunization of rabbits

Thirty-six New Zealand white male rabbits with the mean weight of 2000 ± 200 grams were prepared from Pasteur's Institute. Animals were fed with normal diet (Super Fosskorn) for two weeks and then were divided into three groups of twelve rabbits each and matched for age, body weight, and plasma cholesterol levels. Also, environmental characteristics (light, temperature, and assess to water) were similar. The first (n = 12) group was immunized with (160 μg/kg) homologous MDA-modified LDL (protein) and dissolved in PBS and suspended in an equal volume of Freund's adjuvant. The second group (n = 12) was immunized with (160 μg/kg) homologous cu-LDL (protein) and dissolved in PBS and suspended in an equal volume of the same concentration of Freund's adjuvant. The third group (n = 12) was immunized only with PBS (control group). Immunization was repeated with an identical protocol, again after of 2, 4, 6 and 8 weeks.

### Experimental animals and dietary regimens

At the end of immunization, each group of rabbits was divided into two subgroups with 6 rabbits and each sub-group was fed normal or high cholesterol diets (normal rabbit diet supplemented with 1% cholesterol) for 2 months. Rabbits were kept on a 12 hour day/night cycle and had unrestricted access to water and food *ad libitum*.

### Biochemical Measurments

Blood samples were collected from central ear artery of overnight fasted rabbits before starting experiments and at the end of study. Plasma cholesterol, triglyceride, high density cholesterol (HDL-C), low density cholesterol(LDL-C), and fasting blood sugar levels were determined using an automated enzymatic assay by auto-analyzer Hitachi 902 and using special kits (Diasys, Germany). CRP levels were measured by rabbit CRP Eliza Kit(Rapidbio, USA). ox-LDL antibodies measured with Eliza method (Immunodiagnostica, Germany). Also, external standardization for lipoproteins and fasting blood sugar level was done with the central laboratory of the St. Rafael University Hospital of Leuven in Belgium. The results of two laboratories correlated highly.

Isfahan Cardiovascular Research Center Ethics Committee which is a member of office for human research protections, US department of health and human services, approved the present study, and the animals were handled according to guidelines of Isfahan University of Medical Sciences for Laboratory Animal Sciences for the care and use of laboratory animals.

### Evaluation of atherosclerosis

At the end of study, after anaesthetization of rabbits with pentobarbital 5% and cut of chest each animal was sacrificed and gross anatomic examinations and pathologic investigations were performed on the subject. Then, the right and the left coronary arteries as well as aorta were excised and kept in 10% formalin solution to be used for pathologic evaluation. Tissue specimens were sectioned and prepared using particular histological methods and were assessed by a pathologist with respect to the presence of fatty streaks. For every animal subject and from each tissue specimens, either right coronary artery, left coronary artery or aorta; three consecutive sections were prepared and mounted on one slide. Prepared tissue sections were observed using light microscope. They were all scored on the basis of the scale from zero to four [17].

### Statistical analysis

Results were presented as the mean ± SD. Differences in means of biochemical factors and fatty streak scores between groups were statistically analyzed by non-parametric one-way analysis of variance.

P values < 0.05 were considered statistically significant. A computer program (SPSS 13.0, SPSS Inc. Chicago, IL, USA) was used for statistical analysis.

## Results

**Figure 1:**
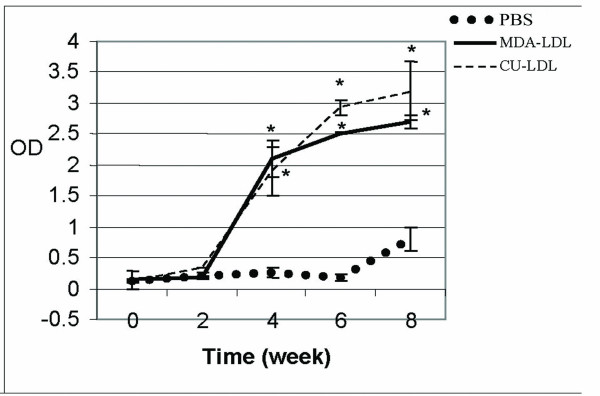
The mean of ox- LDL antibodies in rabbits immunized with MDA-LDL, Cu-LDL, and only PBS in different times. P < 0.05 as compared with rabbits immunized with PBS(Control).

Antibody measurements after 4, 6 and 8 weeks, were statistically more in rabbits immunized with MDA-LDL and Cu-LDL than PBS group (Fig 1).

Table 1 summarized the effect of immunization against MDA-LDL and Cu-LDL on the rabbits fed normal diet or high cholesterol diet on biochemical factors.

**Table 1:** Comparison of the mean of biochemical factors before and after immunization in immunized rabbits fed normal and high cholesterol diet

High cholesterol diet						Normal diet						
Cu-LDL		MDA-LDL		Control		Cu-LDL		MDA-LDL		Control		

After immunization	Before immunization	After immunization	Before immunization	After immunization	Before immunization	After immunization	Before immunization	After immunization	Before immunization	After immunization	Before immunization	Biochemical factor (mg/dl)

503 ± 136.8	18.7 ± 11	403.6 ± 218*	16.8 ± 5.3	558.7 ± 42.2	13.8 ± 8.7	26 ± 15.0	15.3 ± 12.7	21 ± 9.5	19.1 ± 5.7	17.4 ± 11.8	22.2 ± 15.3	LDL-C
128 ± 19.3	39.6 ± 14	114 ± 26.5	35.4 ± 4.9	103 ± 6.2	33.6 ± 1.5	36.7 ± 2.3	29.2 ± 4.9	36.5 ± 3.5	36.5 ± 2.6	30.0 ± 15.3	35.0 ± 8.4	HDL-C
1.2 ± 2.3**	0.8 ± 0.2	2.4 ± 0.3	0.8 ± .24	2.1 ± 0.69	0.9 ± 0.44	1.04 ± 0.5	0.7 ± 0.3	1.1 ± 0.4	0.9 ± 0.25	1.04 ± 0.36	1.04 ± 0.63	CRP
198.8 ± 49.2*	116 ± 20.8	136 ± 41*	105 ± 39.8	254.8 ± 139.4	116 ± 31.4	110 ± 29.6	125.7 ± 19.5	150 ± 10.8	136 ± 16.7	77.0 ± 16.2	63.3 ± 26.6	TG
62.3 ± 3.7*	81.6 ± 9.6	62.3 ± 4.8*	85.4 ± 8.3	82.6 ± 15.7	80.2 ± 39.8	83.7 ± 7.9	58.7 ± 9.9	82 ± 17	58.5 ± 9.9	80.8 ± 11.3	70.0 ± 12.8	FBS
671 ± 144.6*	63.7 ± 15.2	545.8 ± 276.7*	58.8 ± 7.4	711.2 ± 63.4	68.2 ± 15.3	73 ± 18.0	64.7 ± 20.7	93 ± 16.9	55.7 ± 5.3	63.0 ± 27.1	71.0 ± 24.0	CHOL

LDL-C: Low Density Lipoprotein, HDL-C: High Density Lipoprotein, CRP: C-Reactive Protein, TG: Triglyceride, FBS: Fasting Blood Sugar, CHOL: Cholesterol Measurements were expressed as Mean ± SD

* Statistically significant difference with non- immunized high-cholesterol diet (P < 0.05)

** Statistically significant difference with non-immunized high-cholesterol diet and also with high cholesterol diet immunized with MDA-LDL (P < 0.05)

Immunization with MDA-LDL under high cholesterol diet statistically decreased serum concentration of triglyceride, cholesterol, fasting blood sugar, and LDL-C as compared with non-immunized high-cholesterol group (Table 1). Immunization with Cu-LDL under high cholesterol diet statistically decreased serum concentration of triglyceride, cholesterol, fasting blood sugar and CRP as compared with non-immunized high cholesterol group (Table 1). Also, it was a significant difference between the mean changes of fatty streak measurement in aorta and right coronary artery in group under high cholesterol diet immunized with MDA-LDL as compared with non-immunized high cholestrol group (Table 2). In the group under high cholesterol diet immunized with Cu-LDL, the mean of CRP was statistically lower than group under high cholesterol diet immunized with MDA-LDL and also hypercholesterolemic group. At the end of the study in groups under normal diet immunized with MDA-LDL or Cu-LDL, no significant difference in the mean of biochemical factors or fatty streaks was observed (Table 1 and Table 2).

**Table 2:** Comparison of the development of fatty streak in rabbits immunized with MDA-LDL or Cu-LDL fed normal and high cholesterol diet

**Normal diet**			**High cholesterol diet**			
**Cu-LDL**	**MDA-LDL**	**Control**	**Cu-LDL**	**MDA-LDL**	**Control**	**Artery**

0.57 ± 0.32*	0.43 ± 0.42*	0.42 ± 0.37*	3.4 ± 1.7	2.4 ± 1.7 *	3.2 ± 1.5	**Right coronary**
0.1 ± 0.01*	0.5 ± 0.1*	0.5 ± 0.1*	2.8 ± 1.5	2.1 ± 0.5	2.5 ± 1.8	**Left coronary**
0.5 ± 0.1*	0.5 ± 0.1*	0.5 ± 0.1*	3.5 ± 0.05	2.7 ± 0.52*	3.4 ± 0.15	**Aorta**

*Statistically significant difference with non- immunized high-cholesterol diet (P < 0.05)

## Discussion

In the present study, significant decrease in fatty streak in aorta and right coronary arteries in immunized rabbits with MDA-LDL fed high cholesterol diet was observed whereas in rabbits immunized with Cu-LDL fed high cholesterol diet, no significant changes in fatty streaks was found. In Palinski and Ameli studies (on rabbits fed with high cholesterol diet through immunization with MDA-LDL and Cu-LDL, respectively); it was found decrease of atherosclerosis severity [18,19]. In the two distinct studies of Palinski and Ameli different animal models, different antigens, different modes of immunizations, and different techniques for evaluation were used but in our study we tried to evaluate the effects in the same study in addition to use two different dietary regimen. Also, in this study, decreasing of cholesterol, LDL-C, triglyceride and fasting blood sugar in group immunized with MDA-LDL fed high cholesterol diet in comparison with control group and decrease of triglyceride, cholesterol, fasting blood sugar and CRP in group immunized with Cu-LDL fed high cholesterol diet in comparison with control group were found. Therefore, these results can be an expression of differences in the mechanism of these two antigens. In several studies, it was found that CRP as a nonspecific marker for diagnosis of cardiovascular disease can indirectly influence on atherosclerosis [20]. It is suggested to study the effect of immunization on different inflammatory markers and its short and long term effect. Therefore, selection of a suitable antigen can probably influence on increasing of immunization effects on atherosclerosis. Also, in our study, since immunization with MDA-LDL or Cu-LDL did not influence biochemical factors and fatty streaks in normal diet, atherogenic effect of immune system not be proved. In present study, we also used Freund's adjuvant. Administration of Freund's adjuvant alone can reduce formation of mature atherosclerotic lesions in experimental animal [21,22]. Therefore we can probably ascribe a part of our study results to this adjuvant.

## Conclusion

In the present study, we concluded that immune response to ox-LDL is anti atherogenic; Although, the effect of produced antibodies in various methods and dietary regimens was different. Therefore, the hypothesis of the role of protective effects of immune system on atherosclerosis will be strongly debated. As the concept of immunotherapy is in its early stage of development so it should be evaluated and therefore it is logical to study: when immunization should be started which antigen is effective and if dietary regimen or patient condition is effective in decreasing atherosclerosis or not.

## Authors' contributions

S. Asgary carried out and directed the study and conceived it and perform its design also prepared the manuscript and participated in its design and approved the final manuscript.

S. Saberi did laboratory exam.

S. Azampanah participated for data collection.

All of the authors read and approved the final manuscript.
